# Body Mass Index Is Associated with the Severity and All-Cause Mortality of Acute Kidney Injury in Critically Ill Patients: An Analysis of a Large Critical Care Database

**DOI:** 10.1155/2021/6616120

**Published:** 2021-06-28

**Authors:** Benji Wang, Diwen Li, Yuqiang Gong, Binyu Ying, Bihuan Cheng, Laifang Sun

**Affiliations:** ^1^Department of Anesthesiology, Critical Care and Pain Medicine, The Second Affiliated Hospital and Yuying Children's Hospital of Wenzhou Medical University, Wenzhou, 325000 Zhejiang, China; ^2^Department of Emergency Intensive Care Unit, The Second Affiliated Hospital and Yuying Children's Hospital of Wenzhou Medical University, Wenzhou, 325000 Zhejiang, China

## Abstract

**Background:**

Acute kidney injury (AKI) is a common clinical syndrome carrying high morbidity and mortality. Body mass index (BMI) is a common health indicator, and a high BMI value-obesity has been shown to be associated with the outcomes of several diseases. However, the relationship between different BMI categories and mortality in all critically ill patients with AKI is unclear and needs further investigation. Therefore, we evaluated the ability of BMI to predict the severity and all-cause mortality of AKI in critically ill patients.

**Methods:**

We extracted clinical data from the MIMIC-III v1.4 database. All adult patients with AKI were initially screened. The baseline data extracted within 24 hours after ICU admission were presented according to WHO BMI categories. Logistic regression models and the Cox proportional hazards models were, respectively, constructed to assess the relationship between BMI and the severity and all-cause mortality of AKI. The generalized additive model (GAM) was used to identify nonlinear relationships as BMI was a continuous variable. The subgroup analyses were performed to further analyze the stability of the association between BMI category and 365-day all-cause mortality of AKI.

**Result:**

A total of 15,174 patients were extracted and were divided into four groups according to BMI. Obese patients were more likely to be young and male. In the fully adjusted logistic regression model, we found that overweight and obesity were significant predictors of AKI stage III (OR, 95 CI: 1.17, 1.05–1.30; 1.32, 1.18–1.47). In the fully adjusted Cox proportional hazards model, overweight and obesity were associated with significantly lower 30-day, 90-day, and 365-day all-cause mortality. The corresponding adjusted HRs (95 CIs) for overweight patients were 0.87 (0.77, 0.99), 0.84 (0.76, 0.93), and 0.80 (0.74, 0.88), and for obese patients, they were 0.87 (0.77, 0.98), 0.79 (0.71, 0.88), and 0.73 (0.66, 0.80), respectively. The subgroup analyses further presented a stable relationship between BMI category and 365-day all-cause mortality.

**Conclusions:**

BMI was independently associated with the severity and all-cause mortality of AKI in critical illness. Overweight and obesity were associated with increased risk of AKI stage III; however, they were predictive of a relatively lower mortality risk in these patients.

## 1. Introduction

Acute kidney injury (AKI) is a common clinical syndrome carrying high morbidity and mortality [1, 2]. Several studies have shown that AKI occurs in a large number of critically ill patients, especially in the intensive care unit (ICU), carrying a very high mortality rate (50–60%) [3, 4]. Worldwide, 21–67% of critically ill patients develop AKI during their ICU stay [5–7], and the morbidity associated with AKI is expected to continue to increase [8]. Patients with AKI sometimes require renal replacement therapy (RRT); however, clinicians often disagree as to when to start RRT [9]. RRT is an invasive and potentially risky treatment that should not be initiated if the patient has mild disease and kidney function could be restored without intervention. However, a conservative approach to initiation of RRT late in the course of the AKI may lead to adverse outcomes [10]. Therefore, a simple and reliable clinical predictor of AKI severity can better assist clinicians in making decisions.

Body mass index (BMI) is a common health indicator, calculated as the ratio of weight to squared height (kg/m^2^). The degree of obesity can be classified into categories on the basis of BMI according to the World Health Organization (WHO). Overweight (BMI ≥ 25 to <30 kg/m^2^) and obesity (BMI ≥ 30 kg/m^2^) are now common public health problems in both developed and developing countries that give rise to substantial global public burdens [11]. More than half of American adults are overweight or obese, and this proportion is increasing [12, 13]. Studies have shown that obesity is associated with an increased morbidity in cardiovascular disease, diabetes, and depression [14–16], increasing hospital length of stay and medical costs [17, 18].

Previous studies have shown that obese patients have a higher incidence of AKI, more severe renal injury, and higher mortality [19, 20]. Danziger et al. [21] demonstrated that the mortality rate of obese patients with AKI was significantly higher than those without AKI within each BMI category. However, some recent studies have shown that obesity in critically ill patients can improve survival [20, 22]. Soto et al. [23] investigated the association between BMI and AKI in patients with acute respiratory distress syndrome (ARDS) and demonstrated that increased BMI was correlated with increased prevalence of AKI, however, with decreased mortality. In addition, Kim et al. [24] found that in patients with AKI treated with CRRT, a high BMI was beneficial for survival, especially in those with severe illness.

Based on these findings, the relationship between different BMI categories and all-cause mortality in all critically ill patients with AKI is controversial and needs further investigation. Therefore, the aim of this study was to assess the relationship between BMI and the severity and all-cause mortality of AKI in critical illness.

## 2. Methods

### 2.1. Data Source

The Multiparameter Intelligent Monitoring in Intensive Care Database III version 1.4 (MIMIC-III v1.4) includes more than 40,000 ICU patients treated at Beth Israel Deaconess Medical Center (Boston, MA, USA) from 2001 to 2012 [25]. We were allowed to access the database after completing the National Institutes of Health's web-based course and passing the Protecting Human Research Participants exam (No. 6182750). Identifying information was hidden to protect privacy. The project was granted a waiver of informed consent.

### 2.2. Study Population

All adult patients (≥18 years) with AKI according to Kidney Disease Improving Global Outcomes (KDIGO) definition [26] were initially screened. According to the KDIGO classification, AKI was divided into three stages: I, II, and III. Weight and height values were extracted and calculated to obtain BMI. Depending on the WHO BMI classification, patients were divided into four groups: underweight (BMI < 18.5 kg/m^2^), normal (BMI: 18.5 to <25 kg/m^2^), overweight (BMI: 25 to <30 kg/m^2^), and obese (BMI ≥ 30 kg/m^2^).

### 2.3. Data Extraction

As in our previous study [27, 28], we used the PostgreSQL tool (version 9.6) to extract clinical data, including patient demographics, vital signs, laboratory test results, scoring systems, and other clinical variables. Only the data for the first ICU admission of each patient were used, and baseline data were extracted within 24 hours after ICU admission. The vital signs included systolic blood pressure (SBP), diastolic blood pressure (DBP), mean blood pressure (MBP), heart rate, respiratory rate, temperature, and saturation of pulse oxygen (SPO_2_). These values were the averages of the first 24 hours after admission. The comorbidities included congestive heart failure (CHF), cardiac arrhythmias, valvular disease, hypertension, renal disease, liver disease, uncomplicated diabetes, complicated diabetes, metastatic cancer, and coagulopathy. Laboratory measurements included anion gap, bicarbonate, creatinine, chloride, glucose, hematocrit, hemoglobin, platelet, sodium, potassium, lactate, blood urea nitrogen (BUN), white blood cells (WBC), prothrombin time (PT), activated partial thromboplastin time (APTT), and international normalized ratio (INR). Sequential organ failure assessment (SOFA) scores [29] and simplified acute physiology scores II (SAPSII) [30] were calculated for each patient at the time of ICU admission. The endpoints were AKI stage III during ICU stay and 30-day, 90-day, and 365-day all-cause mortality.

### 2.4. Statistical Analysis

Baseline characteristics of all patients were stratified in four groups on the basis of the WHO BMI classification. Continuous variables were expressed as mean ± standard deviation (SD) and were compared using one-way ANOVA across groups. Categorical data were expressed as number or percentage and were compared using the chi-squared test or Fisher's exact test.

Logistic regression models were constructed to determine whether BMI was independently associated with the severity of AKI, and the Cox proportional hazards models were used to evaluate the relationship between BMI and all-cause mortality. The normal-weight group was the reference, and the results were presented as odds ratios (ORs) or hazard ratios (HRs) with 95% confidence intervals (95 CIs). Variables based on epidemiological and biological background were incorporated as potential confounders, and these confounders were adjusted based on a change in effect estimate of >10% when added to the model [31]. Because BMI was a continuous variable, a generalized additive model (GAM) was used to identify nonlinear relationships. We performed subgroup analyses to further analyze the association between BMI category and 365-day all-cause mortality of AKI. Modifications and interactions of subgroups were evaluated using likelihood ratio tests.

The data were analyzed using the R software version 3.42 (http://www.R-project.org, The R Foundation) and EmpowerStats version 2.17.8 (http://www.empowerstats.com, X&Y Solutions, Inc., Boston, MA). *P* < 0.05 was considered statistically significant, and all reported *P* values were two-sided.

## 3. Results

### 3.1. Subject Characteristics

Characteristics of the participants are displayed in Table 1. A total of 15,174 patients who met the inclusion criteria were divided into groups according to BMI. There were four groups: 378 patients were in the underweight group, 4,683 patients were in the normal group, 5,084 patients were in the overweight group, and 5,029 patients were in the obese group. Obese patients were more likely to be young and male; with faster respiratory rate and lower SPO_2_; and with higher anion gap, bicarbonate, creatinine, glucose, BUN, WBC, hematocrit, and hemoglobin levels, as well as higher comorbidities of diabetes compared with normal patients. Furthermore, obese patients had worse SOFA scores, longer length of stay in the ICU, and lower mortality than the normal-BMI patients.

### 3.2. BMI Values and Severity of AKI

**Figure 1:**
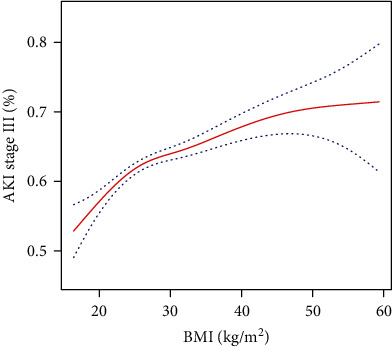
The relationship between BMI values and AKI stage III.

In multivariate logistic regression models (Table 2), adjusted for age, gender, and ethnicity, overweight and obesity were significant predictors of AKI stage III during ICU stay. The corresponding adjusted ORs (95 CIs) for overweight and obese groups compared to the normal group were 1.14 (1.05, 1.24) and 1.28 (1.18, 1.39), respectively. In model II, after adjusting for age, gender, ethnicity, CHF, cardiac arrhythmias, valvular disease, hypertension, uncomplicated diabetes, complicated diabetes, renal failure, liver disease, metastatic cancer, coagulopathy, alcohol abuse, drug abuse, anion gap, bicarbonate, glucose, creatinine, chloride, hemoglobin, lactate, platelet, potassium, APTT, PT, sodium, BUN, WBC, heart rate, SBP, respiratory rate, temperature, SPO_2_, ICU length of stay, SOFA score, and SAPSII score, we found that overweight and obesity remained significant predictors of AKI stage III (OR, 95 CI: 1.17, 1.05–1.30; 1.32, 1.18–1.47, respectively). The underweight group was not independently associated with this clinical endpoint. As a continuous variable, the relationship between admission BMI values and AKI stage III was nearly positively linear (Figure 1).

### 3.3. BMI Values and All-Cause Mortality of AKI

**Figure 2:**
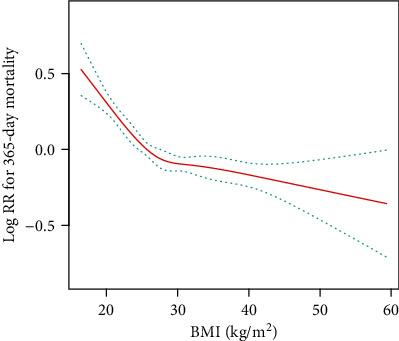
The relationship between BMI values and 365-day mortality of AKI.

Covariates were also adjusted in the Cox proportional hazards models (Table 3). In the fully adjusted model (model II), covariates were adjusted for age, gender, ethnicity, CHF, cardiac arrhythmias, valvular disease, hypertension, uncomplicated diabetes, complicated diabetes, renal failure, liver disease, metastatic cancer, coagulopathy, alcohol abuse, anion gap, bicarbonate, glucose, creatinine, chloride, lactate, potassium, APTT, INR, BUN, WBC, heart rate, SBP, DBP, respiratory rate, temperature, SPO_2_, ICU length of stay, AKI stage, SOFA score, and SAPSII score. Overweight and obesity were associated with significantly lower 30-day, 90-day, and 365-day all-cause mortality than those of the normal group. The corresponding adjusted HRs (95 CIs) for overweight patients were 0.87 (0.77, 0.99), 0.84 (0.76, 0.93), and 0.80 (0.74, 0.88), and those for obese patients were 0.87 (0.77, 0.98), 0.79 (0.71, 0.88), and 0.73 (0.66, 0.80), respectively. Similarly, underweight was not independently associated with all-cause mortality, and BMI was negatively correlated with 365-day all-cause mortality (Figure 2).

### 3.4. Subgroup Analyses

We conducted subgroup analyses to investigate the stability of the associations between BMI category and 365-day all-cause mortality (Table 4). There were no interactions in most strata (*P* = 0.0546‐0.9671). We only observed significant interactions in potassium, lactate, MBP, platelet, SBP, sodium, bicarbonate, and temperature (*P* for interaction: 0.0005, 0.0008, 0.0019, 0.0041, 0.0074, 0.0100, 0.0121, and 0.0173, respectively). However, BMI was a protective factor in all of these interactive stratifications, and as BMI increased, all-cause mortality in the context of AKI decreased.

## 4. Discussion

We included a total of 15,174 patients. In multivariate analysis, after adjusting for age, ethnicity, gender, and other confounding factors, overweight and obesity were associated with increased risk of AKI stage III in critically ill patients, as has previously been reported. However, overweight and obesity were predictive of a relatively lower mortality risk in these patients.

These findings suggest that clinicians should be aware of the possibility of severe AKI in obese patients. Interestingly, obesity is not necessarily an adverse risk factor in critically ill patients with AKI, which is consistent with previously reported survival benefits for critically ill patients with obesity. Previous studies have shown a high morbidity of AKI in critically ill patients during ICU stay [2, 32], while the specific pathogenesis of AKI has remained unclear to date. Obese patients have distinct risk factors for more comorbidities, including hypertension, dyslipidemia, coronary heart disease, and respiratory and cardiovascular failure to present [33–35]. Weight loss after gastric bypass surgery reduced long-term total mortality significantly and particularly improved the prognosis of cancer, heart disease, and diabetes [36, 37]. A retrospective study showed that obesity was associated with increased development of AKI, and increased BMI was associated with decreased mortality in ARDS patients [23]. Another study demonstrated that obesity was associated with short- and long-term survival advantages in patients with sepsis [38]. Our findings in the overweight and obese cohort were in accordance with these two studies.

Studies on the pathophysiological mechanisms between obesity and AKI are limited, but some progress has been made, and the following possible mechanisms are currently believed to exist. Obesity may result in renal compression, leading to increased intrarenal pressure, decreased blood flow to the loop of Henle, and ultimately renal injury [39, 40]. The renin angiotensin aldosterone system (RAAS) in obese people is generally moderately activated, with increased levels of angiotensin II (AngII) and aldosterone [34]. Low levels of AngII increase the reabsorption of sodium by the peritubular capillary, exacerbating the adverse effects of increased blood pressure. AngII increases the hydrostatic pressure of the glomerulus, causing decreased renal perfusion [41]. It has been suggested that there might be an association between leptin and renal injury. Leptin levels tended to be high in obese people, but dropped rapidly after fasting [42]. Patients with chronic kidney disease (CKD) had elevated leptin due to reduce plasma clearance [43]. Studies have shown that these high levels of leptin might in turn lead to faster declines in renal clearance both through the direct effects of nephron disruption and indirect effects through inflammatory responses [44].

Recently, the findings on obesity and prognosis of disease have been challenged, and the obesity paradox has been hotly debated. This paradox is characterized by observations that some adverse outcomes occur less frequently in overweight or obese people than in those with normal weight. Nevertheless, it is unclear whether this phenomenon is definitive, as the underlying mechanisms remain poorly understood. In our final regression model, after adjusting for relevant confounders, the survival benefits of overweight and obese patients remained substantial. Niedziela et al. demonstrated that, in episodes of high catabolic responses in critical illness, the nutritional reserve level of obese people and the corresponding increase of energy supply may be crucial for improving the outcome of AKI [45]. Furthermore, adipocytes release adipokine and inflammatory cytokines, including interleukin-10 and interleukin-18, possibly alleviating harmful immune responses, thereby helping to improve survival in critical illness [46]. Obese patients are more likely to receive attention from medical staff, and because of their predisposition to complications and defective physiological reserves, they tend to be treated earlier and more aggressively [47].

Our study has several limitations. First, as a retrospective single-center analysis, bias was inevitable; this may have affected the results to some extent. Second, we did not evaluate indicators of obesity other than BMI, and whether a high BMI value is necessarily indicative of obesity. These uncertainties may have effects on our conclusions. Third, the weight data used to calculate BMI were actual weight of the first day, which might lead to biases as critically ill related fluid retention could not be ignored. Fourth, because of the lack of renal function data before ICU admission, we were unable to assess the CKD status among patients with AKI. Fifth, we did not consider the timing of AKI. Finally, our study only tested associations, not internal mechanisms; therefore, further studies, are needed to confirm this relationship.

## 5. Conclusions

In this large cohort, we found that BMI was independently associated with severity and all-cause mortality of critically ill patients with AKI. Overweight and obesity were associated with increased risk of AKI stage III; however, they were predictive of a relatively lower mortality risk in these patients.

## Figures and Tables

**Table 1:** Baseline characteristics of participants according to BMI category (*N* = 15174).

Characteristic	BMI (kg/m^2^)				*P* value
	Underweight <18.5	Normal ≥18.5, <25	Overweight ≥25, <30	Obese ≥30	
Clinical parameters (*n*)	378	4683	5084	5029	
BMI (kg/m^2^)	17.63 ± 0.58	22.46 ± 1.73	27.38 ± 1.41	35.91 ± 5.60	<0.001
Age (years)	87.05 ± 71.09	81.42 ± 61.16	74.72 ± 47.22	66.80 ± 31.95	<0.001
Gender, *n* (%)					<0.001
Female	225 (59.52)	2000 (42.71)	1672 (32.89)	2056 (40.88)	
Male	153 (40.48)	2683 (57.29)	3412 (67.11)	2973 (59.12)	
Ethnicity, *n* (%)					0.023
White	260 (68.78)	3296 (70.38)	3658 (71.95)	3622 (72.02)	
Black	46 (12.17)	414 (8.84)	405 (7.97)	452 (8.99)	
Other	72 (19.05)	973 (20.78)	1021 (20.08)	955 (18.99)	
SBP (mmHg)	116 ± 17	116 ± 17	116 ± 16	116 ± 16	0.501
DBP (mmHg)	59 ± 11	59 ± 11	59 ± 10	59 ± 19	0.001
MBP (mmHg)	76 ± 11	77 ± 11	77 ± 11	76 ± 10	0.245
Heart rate (beats/minute)	87 ± 16	86 ± 15	86 ± 15	87 ± 16	0.006
Respiratory rate (beats/minute)	19 ± 4	19 ± 4	18 ± 4	19 ± 4	<0.001
Temperature (°C)	36.7 ± 0.6	36.8 ± 0.7	36.8 ± 0.7	36.9 ± 0.7	<0.001
SPO_2_	98 ± 2.7	98 ± 2.3	97 ± 2.2	97 ± 2.3	<0.001
Comorbidities, *n* (%)					
Congestive heart failure	71 (18.78)	816 (17.42)	766 (15.07)	868 (17.26)	0.003
Cardiac arrhythmias	65 (17.20)	891 (19.03)	824 (16.21)	917 (18.23)	0.002
Valvular disease	28 (7.41)	332 (7.09)	241 (4.74)	263 (5.23)	<0.001
Hypertension	55 (14.55)	749 (15.99)	815 (16.03)	824 (16.38)	0.794
Renal disease	75 (19.84)	907 (19.37)	977 (19.22)	973 (19.35)	0.994
Liver disease	29 (7.67)	352 (7.52)	349 (6.86)	410 (8.15)	0.109
Uncomplicated diabetes	38 (10.05)	784 (16.74)	1084 (21.32)	1556 (30.94)	<0.001
Complicated diabetes	27 (7.14)	303 (6.47)	397 (7.81)	569 (11.31)	<0.001
Metastatic cancer	20 (5.29)	189 (4.04)	179 (3.52)	127 (2.53)	<0.001
Coagulopathy	68 (17.99)	711 (15.18)	740 (14.56)	712 (14.16)	0.145
Laboratory parameters					
Anion gap (mmol/L)	12.94 ± 3.52	12.95 ± 3.47	12.90 ± 3.39	13.13 ± 3.29	<0.001
Bicarbonate (mmol/L)	21.48 ± 5.64	21.61 ± 4.91	21.67 ± 4.48	22.20 ± 4.91	<0.001
Creatinine (mEq/L)	1.38 ± 1.38	1.50 ± 1.66	1.49 ± 1.57	1.56 ± 1.54	<0.001
Chloride (mmol/L)	101.98 ± 7.75	102.15 ± 6.51	102.61 ± 6.17	102.03 ± 6.04	0.079
Glucose (mg/dL)	132.99 ± 40.54	136.06 ± 40.48	141.39 ± 42.31	148.23 ± 45.24	<0.001
Hematocrit	27.93 ± 6.11	27.69 ± 6.17	28.09 ± 6.18	28.68 ± 6.25	<0.001
Hemoglobin (g/dL)	9.35 ± 2.01	9.37 ± 2.12	9.52 ± 2.14	9.67 ± 2.14	<0.001
Platelet (10^9^/L)	211.58 ± 127.66	188.25 ± 112.69	181.20 ± 101.84	188.18 ± 101.33	<0.001
Sodium (mmol/L)	136.16 ± 6.05	135.72 ± 5.07	135.77 ± 4.56	135.96 ± 4.84	0.005
Potassium (mmol/L)	3.72 ± 0.60	3.71 ± 0.56	3.73 ± 0.55	3.79 ± 0.57	<0.001
Lactate (mmol/L)	1.74 ± 0.95	1.76 ± 1.29	1.73 ± 1.27	1.76 ± 1.19	0.130
BUN (mg/dL)	26.59 ± 21.97	26.39 ± 21.27	26.03 ± 20.38	28.21 ± 21.77	<0.001
WBC (10^9^/L)	10.29 ± 6.38	10.40 ± 6.73	10.77 ± 11.30	11.00 ± 6.50	<0.001
PT (seconds)	14.89 ± 3.98	14.94 ± 4.54	14.97 ± 4.79	15.14 ± 4.82	0.002
APTT (seconds)	33.71 ± 13.12	33.56 ± 12.80	32.90 ± 12.48	31.67 ± 10.99	<0.001
INR	1.37 ± 0.54	1.38 ± 0.63	1.38 ± 0.61	1.40 ± 0.65	0.009
Scoring systems					
SOFA	4.92 ± 2.96	5.17 ± 3.19	5.31 ± 3.23	5.57 ± 3.34	<0.001
SAPSII	40.85 ± 13.49	39.71 ± 13.94	38.87 ± 13.80	38.71 ± 14.22	<0.001
Alcohol abuse	15 (3.97)	277 (5.92)	307 (6.04)	317 (6.30)	0.307
Drug abuse	11 (2.91)	166 (3.54)	136 (2.68)	119 (2.37)	0.005
ICU LOS (day)	5.79 ± 6.59	5.96 ± 7.70	6.07 ± 8.14	6.65 ± 8.31	<0.001
AKI stage					<0.001
I	98 (25.93)	1079 (23.04)	1119 (22.01)	920 (18.29)	
II	81 (21.43)	786 (16.78)	749 (14.73)	811 (16.13)	
III	199 (52.65)	2818 (60.18)	3216 (63.26)	3298 (65.58)	
30-day mortality, *n* (%)	71 (18.78)	812 (17.34)	687 (13.51)	667 (13.26)	<0.001
90-day mortality, *n* (%)	123 (32.54)	1155 (24.66)	982 (19.32)	923 (18.35)	<0.001
365-day mortality, *n* (%)	177 (46.83)	1632 (34.85)	1363 (26.81)	1260 (25.05)	<0.001

BMI: body mass index; SBP: systolic blood pressure; DBP: diastolic blood pressure; MBP: mean blood pressure; BUN: blood urea nitrogen; WBC: white blood cell; PT: prothrombin time; APTT: activated partial thromboplastin time; INR: international normalized ratio; SOFA: sequential organ failure assessment; SAPSII: simplified acute physiology score II; ICU: intensive care unit; LOS: length of stay; AKI: acute kidney injury.

**Table 2:** Relationship between BMI and AKI stage III in different models.

Variable	Crude model		Model I		Model II	
	OR (95 CIs)	*P* value	OR (95 CIs)	*P* value	OR (95 CIs)	*P* value
BMI (kg/m^2^)	1.02 (1.01, 1.02)	<0.0001	1.02 (1.02, 1.03)	<0.0001	1.02 (1.02, 1.03)	<0.0001
BMI (category) (kg/m^2^)						
<18.5	0.74 (0.60, 0.91)	0.0042	0.74 (0.60, 0.91)	0.0048	0.78 (0.59, 1.03)	0.0828
≥18.5, <25	1.0 (ref)		1.0 (ref)		1.0 (ref)	
≥25, <30	1.14 (1.05, 1.24)	0.0017	1.14 (1.05, 1.24)	0.0013	1.17 (1.05, 1.30)	0.0055
≥30	1.26 (1.16, 1.37)	<0.0001	1.28 (1.18, 1.39)	<0.0001	1.32 (1.18, 1.47)	<0.0001

OR: odds ratio; CI: confidence interval. Models were derived from logistic multivariate regression models. The crude model was adjusted for none. The Adjust I model was adjusted for age, gender, and ethnicity. The Adjust II model was adjusted for age, gender, ethnicity, congestive heart failure, cardiac arrhythmias, valvular disease, hypertension, uncomplicated diabetes, complicated diabetes, renal failure, liver disease, metastatic cancer, coagulopathy, alcohol abuse, drug abuse, anion gap, bicarbonate, glucose, creatinine, chloride, hemoglobin, lactate, platelet, potassium, APTT, PT, sodium, BUN, WBC, heart rate, systolic blood pressure, respiratory rate, temperature, SPO_2_, ICU length of stay, SAPSII, and SOFA.

**Table 3:** HRs (95 CIs) for mortality across groups of BMI.

Variable	Crude model		Model I		Model II	
	HR (95 CIs)	*P* value	HR (95 CIs)	*P* value	HR (95 CIs)	*P* value
30-day all-cause mortality						
BMI (kg/m^2^)	0.98 (0.98, 0.99)	<0.0001	0.99 (0.98, 0.99)	0.0002	0.99 (0.98, 1.00)	0.0092
BMI (category) (kg/m^2^)						
<18.5	1.16 (0.95, 1.42)	0.1438	1.14 (0.93, 1.39)	0.2147	0.96 (0.75, 1.22)	0.7228
≥18.5, <25	1.0(ref)		1.0(ref)		1.0(ref)	
≥25, <30	0.77 (0.69, 0.85)	<0.0001	0.80 (0.72, 0.89)	<0.0001	0.87 (0.77, 0.99)	0.0287
≥30	0.74 (0.67, 0.82)	<0.0001	0.80 (0.72, 0.88)	<0.0001	0.87 (0.77, 0.98)	0.0250
90-day all-cause mortality						
BMI (kg/m^2^)	0.98 (0.97, 0.98)	<0.0001	0.98 (0.98, 0.99)	<0.0001	0.98 (0.98, 0.99)	<0.0001
BMI (category) (kg/m^2^)						
<18.5	1.31 (1.11, 1.54)	0.0012	1.29 (1.10, 1.52)	0.0021	1.05 (0.86, 1.28)	0.6311
≥18.5, <25	1.0(ref)		1.0(ref)		1.0(ref)	
≥25, <30	0.76 (0.70, 0.83)	<0.0001	0.79 (0.72, 0.86)	<0.0001	0.84 (0.76, 0.93)	0.0012
≥30	0.72 (0.66, 0.78)	<0.0001	0.77 (0.71, 0.84)	<0.0001	0.79 (0.71, 0.88)	<0.0001
365-day all-cause mortality						
BMI (kg/m^2^)	0.97 (0.97, 0.98)	<0.0001	0.98 (0.97, 0.98)	<0.0001	0.98 (0.97, 0.98)	<0.0001
BMI (category) (kg/m^2^)						
<18.5	1.40 (1.23, 1.60)	<0.0001	1.38 (1.21, 1.58)	<0.0001	1.16 (0.98, 1.36)	0.0878
≥18.5, <25	1.0(ref)		1.0(ref)		1.0 (ref)	
≥25, <30	0.73 (0.68, 0.79)	<0.0001	0.76 (0.71, 0.82)	<0.0001	0.80 (0.74, 0.88)	<0.0001
≥30	0.68 (0.63, 0.73)	<0.0001	0.73 (0.68, 0.79)	<0.0001	0.73 (0.66, 0.80)	<0.0001

HR: hazard ratio; CI: confidence interval. Models were derived from the Cox proportional hazards regression models. The crude model was adjusted for none. The Adjust I model was adjusted for age, gender, and ethnicity. The Adjust II model was adjusted for age, gender, ethnicity, congestive heart failure, cardiac arrhythmias, valvular disease, hypertension, uncomplicated diabetes, complicated diabetes, renal failure, liver disease, metastatic cancer, coagulopathy, alcohol abuse, anion gap, bicarbonate, glucose, creatinine, chloride, lactate, potassium, activated partial thromboplastin time, international normalized ratio, blood urea nitrogen, white blood cell, heart rate, systolic blood pressure, diastolic blood pressure, respiratory rate, temperature, SPO_2_, ICU length of stay, AKI stage, SAPSII, and SOFA.

**Table 4:** Subgroup analysis of the associations between BMI category and 365-day all-cause mortality.

	No. of patients	BMI (kg/m^2^)				*P* for interaction
		<18.5	≥18.5, <25	≥25, <30	≥30	
CHF						0.9671
No	12653	1.44 (1.21, 1.73)	1.0 (ref)	0.74 (0.68, 0.81)	0.73 (0.67, 0.79)	
Yes	2521	1.15 (0.85, 1.57)	1.0 (ref)	0.87 (0.75, 0.99)	0.69 (0.60, 0.80)	
Cardiac arrhythmias						0.1640
No	12477	1.47 (1.23, 1.76)	1.0 (ref)	0.74 (0.68, 0.81)	0.72 (0.66, 0.79)	
Yes	2697	1.18 (0.85, 1.62)	1.0 (ref)	0.85 (0.75, 0.97)	0.71 (0.62, 0.81)	
Valvular disease						0.2122
No	14310	1.48 (1.26, 1.73)	1.0 (ref)	0.77 (0.71, 0.83)	0.74 (0.68, 0.80)	
Yes	864	0.61 (0.33, 1.13)	1.0 (ref)	0.87 (0.68, 1.10)	0.71 (0.55, 0.91)	
Hypertension						0.3760
No	12731	1.39 (1.17, 1.65)	1.0 (ref)	0.74 (0.68, 0.81)	0.73 (0.67, 0.80)	
Yes	2443	1.36 (0.95, 1.95)	1.0 (ref)	0.83 (0.71, 0.96)	0.70 (0.59, 0.82)	
Uncomplicated diabetes						0.4680
No	11712	1.37 (1.16, 1.62)	1.0 (ref)	0.78 (0.72, 0.85)	0.77 (0.71, 0.84)	
Yes	3462	1.45 (0.94, 2.23)	1.0 (ref)	0.68 (0.58, 0.80)	0.61 (0.52, 0.71)	
Complicated diabetes						0.5448
No	13878	1.34 (1.14, 1.58)	1.0 (ref)	0.75 (0.70, 0.81)	0.73 (0.67, 0.78)	
Yes	1296	1.93 (1.13, 3.27)	1.0 (ref)	0.86 (0.67, 1.10)	0.74 (0.58, 0.94)	
Metastatic cancer						0.1714
No	14659	1.40 (1.19, 1.64)	1.0 (ref)	0.76 (0.70, 0.82)	0.74 (0.69, 0.80)	
Yes	515	0.87 (0.51, 1.48)	1.0 (ref)	0.82 (0.64, 1.04)	0.82 (0.62, 1.07)	
Coagulopathy						0.1361
No	12943	1.38 (1.16, 1.65)	1.0 (ref)	0.74 (0.68, 0.81)	0.69 (0.63, 0.75)	
Yes	2231	1.32 (0.95, 1.83)	1.0 (ref)	0.88 (0.75, 1.03)	0.97 (0.83, 1.13)	
Renal disease						0.1568
No	12242	1.39 (1.16, 1.67)	1.0 (ref)	0.74 (0.68, 0.81)	0.74 (0.68, 0.81)	
Yes	2932	1.26 (0.92, 1.72)	1.0 (ref)	0.82 (0.72, 0.94)	0.68 (0.59, 0.78)	
Liver disease						0.0794
No	14034	1.40 (1.19, 1.65)	1.0 (ref)	0.76 (0.70, 0.82)	0.71 (0.66, 0.77)	
Yes	1140	1.07 (0.61, 1.90)	1.0 (ref)	0.89 (0.70, 1.12)	0.92 (0.74, 1.16)	
Alcohol abuse						0.1953
No	14258	1.36 (1.16, 1.59)	1.0 (ref)	0.75 (0.70, 0.81)	0.71 (0.66, 0.77)	
Yes	916	1.70 (0.74, 3.95)	1.0 (ref)	0.99 (0.71, 1.38)	1.18 (0.86, 1.63)	
Drug abuse						0.2702
No	14742	1.39 (1.19, 1.63)	1.0 (ref)	0.77 (0.71, 0.82)	0.73 (0.67, 0.78)	
Yes	432	0.60 (0.14, 2.51)	1.0 (ref)	0.56 (0.33, 0.94)	0.80 (0.49, 1.31)	
Heart rate (beats/minute)						0.0775
<85	7600	1.21 (0.95, 1.53)	1.0 (ref)	0.71 (0.64, 0.79)	0.73 (0.65, 0.81)	
≥85	7548	1.52 (1.24, 1.87)	1.0 (ref)	0.82 (0.74, 0.91)	0.74 (0.67, 0.82)	
SBP (mmHg)						0.0074
<114	7585	1.22 (0.98, 1.51)	1.0 (ref)	0.81 (0.74, 0.90)	0.78 (0.71, 0.86)	
≥114	7551	1.56 (1.25, 1.95)	1.0 (ref)	0.70 (0.62, 0.78)	0.67 (0.60, 0.75)	
DBP (mmHg)						0.2996
<58	7595	1.27 (1.02, 1.58)	1.0 (ref)	0.82 (0.74, 0.90)	0.79 (0.71, 0.87)	
≥58	7540	1.48 (1.19, 1.84)	1.0 (ref)	0.70 (0.62, 0.78)	0.67 (0.60, 0.74)	
MBP (mmHg)						0.0019
<75	7591	1.27 (1.02, 1.58)	1.0 (ref)	0.82 (0.74, 0.90)	0.78 (0.71, 0.87)	
≥75	7557	1.49 (1.19, 1.85)	1.0 (ref)	0.70 (0.63, 0.78)	0.67 (0.60, 0.75)	
Respiratory rate (beats/minute)						0.0546
<18	7607	1.41 (1.10, 1.80)	1.0 (ref)	0.71 (0.63, 0.79)	0.73 (0.65, 0.82)	
≥19	7531	1.32 (1.08, 1.61)	1.0 (ref)	0.80 (0.73, 0.88)	0.69 (0.63, 0.76)	
Temperature (°C)						0.0173
<36.8	7315	1.27 (1.04, 1.54)	1.0 (ref)	0.76 (0.69, 0.84)	0.77 (0.70, 0.85)	
≥36.8	7326	1.56 (1.20, 2.02)	1.0 (ref)	0.78 (0.70, 0.88)	0.71 (0.63, 0.80)	
SPO_2_						0.1292
<98	7547	1.24 (0.98, 1.56)	1.0 (ref)	0.71 (0.64, 0.78)	0.63 (0.57, 0.69)	
≥98	7599	1.54 (1.25, 1.90)	1.0 (ref)	0.80 (0.72, 0.89)	0.80 (0.71, 0.90)	
Anion gap (mmol/L)						0.2197
<13	7128	1.62 (1.28, 2.04)	1.0 (ref)	0.68 (0.61, 0.77)	0.69 (0.61, 0.79)	
≥13	7551	1.27 (1.02, 1.57)	1.0 (ref)	0.81 (0.74, 0.89)	0.72 (0.66, 0.79)	
Bicarbonate (mg/dL)						0.0121
<22	6609	1.20 (0.97, 1.49)	1.0 (ref)	0.82 (0.74, 0.90)	0.82 (0.74, 0.91)	
≥22	8470	1.58 (1.26, 1.98)	1.0 (ref)	0.71 (0.64, 0.79)	0.67 (0.60, 0.74)	
Creatinine (mEq/L)						0.4265
<1.0	6950	1.68 (1.32, 2.14)	1.0 (ref)	0.72 (0.64, 0.82)	0.65 (0.56, 0.74)	
≥1.0	8188	1.21 (0.99, 1.48)	1.0 (ref)	0.74 (0.68, 0.81)	0.67 (0.61, 0.73)	
BUN (mg/dL)						0.6121
<20	7467	1.83 (1.41, 2.36)	1.0 (ref)	0.71 (0.62, 0.81)	0.64 (0.56, 0.74)	
≥20	7669	1.13 (0.93, 1.38)	1.0 (ref)	0.78 (0.71, 0.85)	0.70 (0.64, 0.76)	
WBC (10^9^/L)						0.8296
<9.6	7477	1.35 (1.09, 1.68)	1.0 (ref)	0.74 (0.67, 0.82)	0.74 (0.66, 0.82)	
≥9.6	7587	1.43 (1.14, 1.79)	1.0 (ref)	0.77 (0.70, 0.86)	0.71 (0.65, 0.79)	
Hematocrit						0.1928
<27.8	7577	1.57 (1.26, 1.94)	1.0 (ref)	0.79 (0.71, 0.87)	0.79 (0.71, 0.88)	
≥27.8	7562	1.19 (0.95, 1.49)	1.0 (ref)	0.74 (0.66, 0.81)	0.67 (0.61, 0.74)	
Hemoglobin (g/dL)						0.1746
<9.4	7558	1.51 (1.23, 1.86)	1.0 (ref)	0.78 (0.70, 0.86)	0.77 (0.69, 0.85)	
≥9.4	7565	1.21 (0.95, 1.52)	1.0 (ref)	0.75 (0.67, 0.83)	0.69 (0.62, 0.77)	
Platelet (10^9^/L)						0.0041
<169	7599	1.35 (1.06, 1.71)	1.0 (ref)	0.73 (0.66, 0.81)	0.80 (0.72, 0.88)	
≥169	7497	1.41 (1.14, 1.73)	1.0 (ref)	0.80 (0.72, 0.89)	0.67 (0.60, 0.75)	
Potassium (mmol/L)						0.0005
<3.7	6825	1.45 (1.15, 1.84)	1.0 (ref)	0.79 (0.70, 0.88)	0.85 (0.76, 0.95)	
≥3.7	8322	1.32 (1.07, 1.63)	1.0 (ref)	0.74 (0.67, 0.81)	0.64 (0.58, 0.70)	
Sodium (mmol/L)						0.0100
<136	6723	1.32 (1.04, 1.67)	1.0 (ref)	0.82 (0.74, 0.91)	0.78 (0.70, 0.88)	
≥136	8422	1.40 (1.14, 1.72)	1.0 (ref)	0.72 (0.65, 0.79)	0.69 (0.62, 0.76)	
Chloride (mmol/L)						0.2410
<103	7245	1.29 (1.05, 1.59)	1.0 (ref)	0.80 (0.73, 0.88)	0.70 (0.64, 0.78)	
≥103	7857	1.43 (1.13, 1.81)	1.0 (ref)	0.75 (0.67, 0.83)	0.76 (0.68, 0.85)	
PT (seconds)						0.3228
<13.9	6958	1.41 (1.11, 1.80)	1.0 (ref)	0.71 (0.64, 0.80)	0.62 (0.55, 0.71)	
≥13.9	7255	1.30 (1.04, 1.63)	1.0 (ref)	0.79 (0.71, 0.87)	0.79 (0.72, 0.87)	
APTT (seconds)						0.2013
<30	7046	1.35 (1.04, 1.74)	1.0 (ref)	0.76 (0.68, 0.85)	0.67 (0.60, 0.76)	
≥30	7144	1.36 (1.10, 1.68)	1.0 (ref)	0.76 (0.69, 0.84)	0.83 (0.75, 0.92)	
INR						0.1598
<1.2	4638	1.36 (1.03, 1.81)	1.0 (ref)	0.67 (0.58, 0.77)	0.58 (0.49, 0.67)	
≥1.2	9576	1.36 (1.12, 1.67)	1.0 (ref)	0.79 (0.73, 0.86)	0.78 (0.72, 0.86)	
Lactate (mmol/L)						0.0008
<1.4	4791	1.58 (1.19, 2.11)	1.0 (ref)	0.71 (0.62, 0.81)	0.62 (0.53, 0.71)	
≥1.4	5768	1.26 (1.00, 1.58)	1.0 (ref)	0.87 (0.78, 0.97)	0.84 (0.75, 0.93)	
Glucose (mg/dL)						0.4116
<132	7567	1.49 (1.22, 1.84)	1.0 (ref)	0.68 (0.61, 0.76)	0.70 (0.62, 0.78)	
≥132	7553	1.25 (0.99, 1.59)	1.0 (ref)	0.81 (0.74, 0.90)	0.72 (0.65, 0.79)	
SOFA score						0.5211
<5	6918	1.49 (1.18, 1.89)	1.0 (ref)	0.78 (0.69, 0.88)	0.61 (0.54, 0.70)	
≥5	8256	1.34 (1.09, 1.64)	1.0 (ref)	0.74 (0.68, 0.81)	0.75 (0.69, 0.82)	
SAPSII score						0.0768
<37	7070	1.75 (1.30, 2.35)	1.0 (ref)	0.72 (0.62, 0.83)	0.67 (0.58, 0.77)	
≥37	8104	1.22 (1.02, 1.47)	1.0 (ref)	0.79 (0.73, 0.86)	0.77 (0.70, 0.84)	

CHF: congestive heart failure; SBP: systolic blood pressure; DBP: diastolic blood pressure; MBP: mean blood pressure; BUN: blood urea nitrogen; WBC: white blood cell; PT: prothrombin time; APTT: activated partial thromboplastin time; INR: international normalized ratio; SOFA: sequential organ failure assessment; SAPSII: simplified acute physiology score II. HRs (95 CIs) were derived from the Cox proportional hazards regression models.

## Data Availability

The clinical data used to support the findings of this study were supplied by the Multiparameter Intelligent Monitoring in Intensive Care Database III version 1.4 (MIMIC-III v.1.4). Although the database is publicly and freely available, researchers must complete the National Institutes of Health's web-based course known as Protecting Human Research Participants to apply for permission to access the database.

## Supplementary Materials

Supplementary Materials STable 1: baseline characteristics of participants according to AKI classification. BMI: body mass index; SBP: systolic blood pressure; DBP: diastolic blood pressure; MBP: mean blood pressure; BUN: blood urea nitrogen; WBC: white blood cell; PT: prothrombin time; APTT: activated partial thromboplastin time; INR: international normalized ratio; SOFA: sequential organ failure assessment; SAPSII: simplified acute physiology score II; ICU: intensive care unit; LOS: length of stay; AKI: acute kidney injury.

## References

[1] Kanagasundaram N. S. (2015). Pathophysiology of ischaemic acute kidney injury. *Annals of Clinical Biochemistry*.

[2] Uchino S., Kellum J. A., Bellomo R. (2005). Acute renal failure in critically ill patients: a multinational, multicenter study. *JAMA*.

[3] Hofhuis J. G., van Stel H. F., Schrijvers A. J., Rommes J. H., Spronk P. E. (2013). The effect of acute kidney injury on long-term health-related quality of life: a prospective follow-up study. *Critical care (London, England)*.

[4] White L. E., Hassoun H. T., Bihorac A. (2013). Acute kidney injury is surprisingly common and a powerful predictor of mortality in surgical sepsis. *The journal of trauma and acute care surgery*.

[5] Hoste E. A., Clermont G., Kersten A. (2006). RIFLE criteria for acute kidney injury are associated with hospital mortality in critically ill patients: a cohort analysis. *Critical care (London, England)*.

[6] Hong S. E., Kim T. Y., Yoo J. H. (2017). Acute kidney injury can predict in-hospital and long-term mortality in elderly patients undergoing hip fracture surgery. *PLoS One*.

[7] Hoste E. A., Bagshaw S. M., Bellomo R. (2015). Epidemiology of acute kidney injury in critically ill patients: the multinational AKI-EPI study. *Intensive Care Medicine*.

[8] Sawhney S., Fraser S. D. (2017). Epidemiology of AKI: utilizing large databases to determine the burden of AKI. *Advances in Chronic Kidney Disease*.

[9] Xiao L., Jia L., Li R., Zhang Y., Ji H., Faramand A. (2019). Early versus late initiation of renal replacement therapy for acute kidney injury in critically ill patients: a systematic review and meta-analysis. *PLoS One*.

[10] Digvijay K., Neri M., Fan W., Ricci Z., Ronco C. (2019). International survey on the management of acute kidney injury and continuous renal replacement therapies: year 2018. *Blood Purification*.

[11] Ng M., Fleming T., Robinson M. (2014). Global, regional, and national prevalence of overweight and obesity in children and adults during 1980-2013: a systematic analysis for the Global Burden of Disease Study 2013. *Lancet*.

[12] Ogden C. L., Carroll M. D., Lawman H. G. (2016). Trends in obesity prevalence among children and adolescents in the United States, 1988-1994 through 2013-2014. *JAMA*.

[13] Perak A. M., Ning H., Kit B. K. (2019). Trends in levels of lipids and apolipoprotein B in US youths aged 6 to 19 years, 1999-2016. *JAMA*.

[14] Barton B. B., Zagler A., Engl K., Rihs L., Musil R. (2020). Prevalence of obesity, metabolic syndrome, diabetes and risk of cardiovascular disease in a psychiatric inpatient sample: results of the Metabolism in Psychiatry (MiP) Study. *European Archives of Psychiatry and Clinical Neuroscience*.

[15] Sorop O., Olver T. D., van de Wouw J. (2017). The microcirculation: a key player in obesity-associated cardiovascular disease. *Cardiovascular Research*.

[16] Madadi F., Jawad R., Mousati I., Plaeke P., Hubens G. (2019). Remission of type 2 diabetes and sleeve gastrectomy in morbid obesity: a comparative systematic review and meta-analysis. *Obesity Surgery*.

[17] Kremers H. M., Visscher S. L., Kremers W. K., Naessens J. M., Lewallen D. G. (2014). Obesity increases length of stay and direct medical costs in total hip arthroplasty. *Clinical Orthopaedics and Related Research*.

[18] Freckelton L., Lambert K., Smith N. A., Westley-Wise V., Lago L., Mullan J. (2019). Impact of body mass index on utilization of selected hospital resources for four common surgical procedures. *ANZ Journal of Surgery*.

[19] Bercault N., Boulain T., Kuteifan K., Wolf M., Runge I., Fleury J.-C. (2004). Obesity-related excess mortality rate in an adult intensive care unit: a risk-adjusted matched cohort study. *Critical Care Medicine*.

[20] Druml W., Metnitz B., Schaden E., Bauer P., Metnitz P. G. H. (2010). Impact of body mass on incidence and prognosis of acute kidney injury requiring renal replacement therapy. *Intensive Care Medicine*.

[21] Danziger J., Chen K. P., Lee J. (2016). Obesity, acute kidney injury, and mortality in critical illness. *Critical Care Medicine*.

[22] Pickkers P., de Keizer N., Dusseljee J., Weerheijm D., van der Hoeven J. G., Peek N. (2013). Body mass index is associated with hospital mortality in critically ill patients: an observational cohort study. *Critical Care Medicine*.

[23] Soto G. J., Frank A. J., Christiani D. C., Gong M. N. (2012). Body mass index and acute kidney injury in the acute respiratory distress syndrome. *Critical Care Medicine*.

[24] Kim H., Kim H., Lee M. (2018). The impact of disease severity on paradoxical association between body mass index and mortality in patients with acute kidney injury undergoing continuous renal replacement therapy. *BMC Nephrology*.

[25] Johnson A. E., Pollard T. J., Shen L. (2016). MIMIC-III, a freely accessible critical care database. *Scientific data*.

[26] The ad-hoc working group of ERBP, Fliser D., Laville M. (2012). A European Renal Best Practice (ERBP) position statement on the Kidney Disease Improving Global Outcomes (KDIGO) clinical practice guidelines on acute kidney injury: part 1: definitions, conservative management and contrast-induced nephropathy. *Nephrology, dialysis, transplantation : official publication of the European Dialysis and Transplant Association - European Renal Association*.

[27] Wang B., Li D., Gong Y., Ying B., Cheng B. (2019). Association of serum total and ionized calcium with all-cause mortality in critically ill patients with acute kidney injury. *Clinica Chimica Acta*.

[28] Wang B., Gong Y., Ying B., Cheng B. (2019). Relation between red cell distribution width and mortality in critically ill patients with acute respiratory distress syndrome. *BioMed Research International*.

[29] Vincent J. L., Moreno R., Takala J. (1996). The SOFA (Sepsis-related Organ Failure Assessment) score to describe organ dysfunction/failure. *Intensive Care Medicine*.

[30] Le Gall J. R., Lemeshow S., Saulnier F. (1993). A new Simplified Acute Physiology Score (SAPS II) based on a European/North American multicenter study. *JAMA*.

[31] Vandenbroucke J. P., von Elm E., Altman D. G. (2014). Strengthening the Reporting of Observational Studies in Epidemiology (STROBE): explanation and elaboration. *International journal of surgery (London, England)*.

[32] Anzueto A., Frutos-Vivar F., Esteban A. (2011). Influence of body mass index on outcome of the mechanically ventilated patients. *Thorax*.

[33] Lavie C. J., Milani R. V., Ventura H. O. (2009). Obesity and cardiovascular disease: risk factor, paradox, and impact of weight loss. *Journal of the American College of Cardiology*.

[34] Hall J. E., do Carmo J. M., da Silva A. A., Wang Z., Hall M. E. (2015). Obesity-induced hypertension: interaction of neurohumoral and renal mechanisms. *Circulation Research*.

[35] Anari R., Amani R., Latifi S. M., Veissi M., Shahbazian H. (2017). Association of obesity with hypertension and dyslipidemia in type 2 diabetes mellitus subjects. *Diabetes & metabolic syndrome*.

[36] Schauer P. R., Bhatt D. L., Kirwan J. P. (2014). Bariatric surgery versus intensive medical therapy for diabetes—3-year outcomes. *The New England Journal of Medicine*.

[37] Adams T. D., Gress R. E., Smith S. C. (2007). Long-term mortality after gastric bypass surgery. *The New England Journal of Medicine*.

[38] Li S., Hu X., Xu J. (2019). Increased body mass index linked to greater short- and long-term survival in sepsis patients: a retrospective analysis of a large clinical database. *International Journal of Infectious Diseases*.

[39] Hall J. E., Crook E. D., Jones D. W., Wofford M. R., Dubbert P. M. (2002). Mechanisms of obesity-associated cardiovascular and renal disease. *The American Journal of the Medical Sciences*.

[40] Hall J. E., do Carmo J. M., da Silva A. A., Wang Z., Hall M. E. (2019). Obesity, kidney dysfunction and hypertension: mechanistic links. *Nature Reviews Nephrology*.

[41] Kim E. S., Kim H. J., Kim Y. J. (2013). Resistive index as a predictor of acute kidney injury caused by an angiotensin converting enzyme inhibitor or angiotensin II receptor blocker in chronic kidney disease patients. *Kidney research and clinical practice*.

[42] Ahima R. S. (2008). Revisiting leptin's role in obesity and weight loss. *The Journal of Clinical Investigation*.

[43] Mak R. H., Cheung W., Cone R. D., Marks D. L. (2006). Leptin and inflammation-associated cachexia in chronic kidney disease. *Kidney International*.

[44] Ambarkar M., Pemmaraju S. V., Gouroju S. (2016). Adipokines and their relation to endothelial dysfunction in patients with chronic kidney disease. *Journal of clinical and diagnostic research : JCDR*.

[45] Niedziela J., Hudzik B., Niedziela N. (2014). The obesity paradox in acute coronary syndrome: a meta-analysis. *European Journal of Epidemiology*.

[46] McLaughlin T., Deng A., Yee G. (2010). Inflammation in subcutaneous adipose tissue: relationship to adipose cell size. *Diabetologia*.

[47] O’Brien J. M., Philips G. S., Ali N. A., Aberegg S. K., Marsh C. B., Lemeshow S. (2012). The association between body mass index, processes of care, and outcomes from mechanical ventilation: a prospective cohort study. *Critical Care Medicine*.

