# Supplementary material for: Body Mass Index Is Associated with the Severity and All-Cause Mortality of Acute Kidney Injury in Critically Ill Patients: An Analysis of a Large Critical Care Database
Source: Biomed Res Int. 2021 Jun 28;2021:6616120. doi: 10.1155/2021/6616120 (PMC8260311; doi:10.1155/2021/6616120)
Supplement: Supplementary Materials — STable 1: baseline characteristics of participants according to AKI classification. BMI: body mass index; SBP: systolic blood pressure; DBP: diastolic blood pressure; MBP: mean blood pressure; BUN: blood urea nitrogen; WBC: white blood cell; PT: prothrombin time; APTT: activated partial thromboplastin time; INR: international normalized ratio; SOFA: sequential organ failure assessment; SAPSII: simplified acute physiology score II; ICU: intensive care unit; LOS: length of stay; AKI: acute kidney injury. [file 6616120.f1.zip › Supplemental .docx]

**STable 1** Baseline characteristics of participants according to AKI classification

| Characteristic | I and II | III | *P* value |
| --- | --- | --- | --- |
| Clinical parameters, n | 5759 | 9723 |  |
| BMI, kg/m^2^ | 27.9 ± 6.5 | 28.8 ± 6.8 | <0.001 |
| Age, years | 64.2 ± 17.0 | 66.8 ± 15.0 | <0.001 |
| Gender, n (%) |  |  | < 0.279 |
| Female | 2305 (40.0) | 3806 (39.1) |  |
| Male | 3454 (60.0) | 5917 (60.9) |  |
| Ethnicity, n (%) |  |  | <0.001 |
| White | 4114 (71.4) | 6932 (71.3) |  |
| Black | 564 (9.8) | 801 (8.2) |  |
| Other | 1081 (18.8) | 1990 (20.5) |  |
| SBP, mmHg | 117.7 ± 17.0 | 115.5 ± 15.7 | <0.001 |
| DBP, mmHg | 60.7 ± 11.0 | 58.4 ± 10.1 | <0.001 |
| MBP, mmHg | 77.5 ± 11.2 | 76.1 ± 10.3 | <0.001 |
| Heart rate, beats/minute | 86.9 ± 15.9 | 86.2 ± 15.1 | 0.005 |
| Respiratory rate, beats/minute | 19.0 ± 4.2 | 18.6 ± 4.0 | <0.001 |
| Temperature, ℃ | 36.9 ± 0.7 | 36.8 ± 0.7 | <0.001 |
| SPO2, | 97.2 ± 2.4 | 97.4 ± 2.2 | <0.001 |
| Comorbidities, n (%) |  |  |  |
| Congestive heart failure | 1061 (18.4) | 1543 (15.9) | <0.001 |
| Cardiac arrhythmias | 1123 (19.5) | 1628 (16.7) | <0.001 |
| Valvular disease | 365 (6.3) | 519 (5.3) | 0.010 |
| Hypertension | 839 (14.6) | 1655 (17.0) | <0.001 |
| Renal disease | 998 (17.3) | 1996 (20.5) | <0.001 |
| Liver disease | 435 (7.6) | 727 (7.5) | 0.862 |
| Uncomplicated diabetes | 1200 (20.8) | 2325 (23.9) | <0.001 |
| Complicated diabetes | 462 (8.0) | 865 (8.9) | <0.060 |
| Metastatic cancer | 217 (3.8) | 305 (3.1) | 0.035 |
| Coagulopathy | 786 (13.6) | 1485 (15.3) | 0.006 |
| Laboratory parameters |  |  |  |
| Anion gap, mmol/L | 12.8 ± 3.3 | 13.1 ± 3.5 | <0.001 |
| Bicarbonate, mmol/L | 22.1 ± 4.9 | 21.6 ± 4.7 | <0.001 |
| Creatinine, me/L | 1.4 ± 1.6 | 1.6 ± 1.6 | <0.001 |
| Chloride, mmol/L | 102.1 ± 6.6 | 102.3 ± 6.2 | 0.074 |
| Hematocrit, % | 28.9 ± 6.3 | 27.8 ± 6.1 | <0.001 |
| Hemoglobin, g/dL | 9.8 ± 2.2 | 9.4 ± 2.1 | 0.008 |
| Platelet, 10^9^ /L | 197.6 ± 109.0 | 181.3 ± 104.1 | <0.001 |
| Sodium, mmol/L | 136.2 ± 5.0 | 135.6 ± 4.8 | <0.001 |
| Potassium, mmol/L | 3.7 ± 0.6 | 3.7 ± 0.6 | 0.042 |
| Lactate, mmol/L | 1.7 ± 1.2 | 1.8 ± 1.3 | 0.008 |
| BUN, mg/dL | 25.0 ± 19.2 | 28.0 ± 22.2 | <0.001 |
| WBC, 10^9^ /L | 10.6 ± 7.2 | 10.8 ± 9.1 | 0.168 |
| PT, second | 15.0 ± 4.7 | 15.1 ± 4.7 | 0.197 |
| APTT, second | 32.1 ± 12.8 | 33.1 ± 11.8 | <0.001 |
| INR | 1.4 ± 0.6 | 1.4 ± 0.7 | 0.006 |
| Scoring systems |  |  |  |
| SOFA | 4.9 ± 3.1 | 5.6 ± 3.3 | <0.001 |
| SAPSII | 37.5 ± 13.8 | 40.0 ± 14.0 | <0.001 |
| Alcohol abuse | 398 (6.9) | 534 (5.5) | <0.001 |
| Drug abuse | 208 (3.6) | 230 (2.4) | <0.001 |
| ICU LOS, day | 7.3 ± 8.8 | 5.6 ± 7.5 | <0.001 |
| 30-day mortality, n (%) | 783 (13.6) | 1505 (15.5) | <0.001 |
| 90-day mortality, n (%) | 1179 (20.5) | 2080 (21.4) | 0.175 |
| 365-day mortality, n (%) | 1701 (29.5) | 2844 (29.3) | 0.706 |

BMI: body mass index; SBP: systolic blood pressure; DBP: diastolic blood pressure; MBP: mean blood pressure; BUN: blood urea nitrogen; WBC: white blood cell; PT: prothrombin time; APTT: activated partial thromboplastin time; INR: international normalized ratio; SOFA: Sequential Organ Failure Assessment; SAPSII: Simplified Acute Physiology Score II; ICU: intensive care unit; LOS: length of stay; AKI: acute kidney injury.
