# Supplementary material for: Body Mass Index Is Associated with the Severity and All-Cause Mortality of Acute Kidney Injury in Critically Ill Patients: An Analysis of a Large Critical Care Database
Source: Biomed Res Int. 2021 Jun 28;2021:6616120. doi: 10.1155/2021/6616120 (PMC8260311; doi:10.1155/2021/6616120)
Supplement: Supplementary Materials — STable 1: baseline characteristics of participants according to AKI classification. BMI: body mass index; SBP: systolic blood pressure; DBP: diastolic blood pressure; MBP: mean blood pressure; BUN: blood urea nitrogen; WBC: white blood cell; PT: prothrombin time; APTT: activated partial thromboplastin time; INR: international normalized ratio; SOFA: sequential organ failure assessment; SAPSII: simplified acute physiology score II; ICU: intensive care unit; LOS: length of stay; AKI: acute kidney injury. [file 6616120.f1.zip › supplementay description.docx]

**Supplementary Material:**

**STable 1** Baseline characteristics of participants according to AKI classification

BMI: body mass index; SBP: systolic blood pressure; DBP: diastolic blood pressure; MBP: mean blood pressure; BUN: blood urea nitrogen; WBC: white blood cell; PT: prothrombin time; APTT: activated partial thromboplastin time; INR: international normalized ratio; SOFA: Sequential Organ Failure Assessment; SAPSII: Simplified Acute Physiology Score II; ICU: intensive care unit; LOS: length of stay; AKI: acute kidney injury.
